# Babesiosis in cattle and ixodid tick distribution in Dasenech and Salamago Districts, southern Ethiopia

**DOI:** 10.1038/s41598-022-10416-4

**Published:** 2022-04-16

**Authors:** Haben Fesseha, Mesfin Mathewos, Eyob Eshetu, Bereket Tefera

**Affiliations:** 1 School of Veterinary Medicine, Wolaita Sodo University, Wolaita Sodo, Ethiopia; 2 Jinka Town Municipal Abattoir Animal Health Team Leader, South Omo Zone, Jinka, Southern Ethiopia Ethiopia

**Keywords:** Diseases, Health occupations

## Abstract

Babesiosis, caused by protozoan parasites of the genus Babesia, and ixodid ticks are considered to be one of the most important causes that result in significant economic losses in cattle production worldwide, including in Ethiopia. A cross-sectional study was undertaken in the Dasenech and Salamago Districts of South Omo zone Ethiopia to determine the prevalence and associated risk factors of babesia infection and ixodid ticks of cattle using Giemsa-stained thin and thick film techniques and morphological identification keys for babesia species and tick identification, respectively. Out of 470 examined cattle, 102 (21.7%) were infected by Babesiosis (15.53% *Babesia bigemina* and 6.17% *Babesia bovis*). A statistically significant association (*p* < 0.05) was observed between babesia infection and season and tick infestation. However, cattle that were infected with the *Babesia* parasite revealed a lower mean PCV value (21.49%) than noninfected cattle (28.29%) and showed a statistically significant (*p* < 0.05) difference with the occurrence of Babesia infection. The overall prevalence of ixodid ticks was 53.8% (253/470) and revealed a statistically significant association (*p* < 0.05) between the season and origin of the animal. However, no statistically significant association (*p* > 0.05) was observed between sex, age, and body condition score of the animal with the occurrence of ixodid tick. A total of 8040 adult ticks belonging to four tick genera, Amblyomma, Rhipicephalus (Boophilus), Hyalomma, and Rhipicephalus, were collected from various body parts and identified. The high prevalence of Babesia infection and ixodid ticks in cattle at the study sites requires seroprevalence and molecular studies to identify the predominant Babesia species and to detect Babesia in tick hemolymph for the identification of tick genera responsible for the occurrence of Babesia infection. Additionally, tailoring suitable and coordinated tick management methods using chemotherapy as well as strategic treatment to overt clinical cases of bovine babesiosis is critical.

## Introduction

Ethiopia has one of the largest livestock populations in Africa, and livestock production is critical to the country's agricultural development. For instance, there are approximately 56.1 million heads of cattle in Ethiopia, representing a huge population of animals in Africa^1^. Despite the importance of animal production to the economy as a whole, this subsector remains underutilized due to illness, poor husbandry practices, poor genetic makeup, malnutrition, harsh environments, and lack of market infrastructure^2–4^.

Animal illnesses are a significant factor influencing productivity and negatively impacting animal health. Infestations of parasites are strongly linked to the presence and dispersion of their vectors. *Babesia, Trypanosome, Theileria,* and *Anaplasma* species cause bovine hemoparasitic infection, which is spread by arthropods. In tropical and subtropical regions of the world, including Ethiopia, these infectious blood parasites are economically significant vector-borne infections^5,6^.

Babesiosis is the second most common tick-borne disease of mammals after trypanosomiasis^7^ and causes significant morbidity and death in cattle in tropical and subtropical areas^8^. It is caused by protozoan parasites of the genus Babesia, order Piroplasmida, phylum Apicomplexa, and subclass Piroplamsia and is mostly referred to as ‘piroplasmas’ due to the pear-like formed merozoites. Morbidity and mortality are highly variable and are impacted by a variety of factors^8^, including the current treatments used in the area, previous exposure to a parasite species/strain, age, cattle breed, vaccination status, location, sex, herd size, seasonal management, bug abundance, feed density, domestic pet in the household, tick infestations in cattle and barn, and grazing area management^9^.

More than a hundred Babesia species have been identified, infecting a wide range of mammalian hosts; of these, 18 cause significant disease in domestic animals, including cattle, sheep, goats, horses, pigs, dogs, and cats^10^. Cattle are the primary hosts and reservoirs for *B. bovis, B. bigemina, Babesia divergens,* and *Babesia major*^11^. The two most prevalent Babesia species are *B. bovis* and *B. bigemina*, which are found primarily in tropical and subtropical climates, particularly in Asia, Africa, Central and South America, areas of southern Europe, and Australia^4^. Even though *B. bovis* and *B. bigemina* are both found in the same geographic location, their distributions are very different. *B. bigemina* is more widely spread in Africa than *B. bovis*^12^.

Babesiosis is characterized by fever (> 40 °C), which can be high, and varying degrees of hemolysis and anemia. Anemia may develop rapidly and result in clinical signs, including pale mucous membranes, inappetence, a drop in milk production, weakness, lethargy, and increased respiratory and heart rates. Moreover, abortion in pregnant cows and temporarily decreased fertility in bulls are other signs related to anemia or fever. Jaundice is sometimes apparent, especially when the clinical signs are less acute, and hemoglobinuria and hemoglobinemia are common in animals infected with *B. bigemina*. *B. bovis* can cause additional clinical signs via changes in red blood cells (RBCs) that result in their accumulation in capillaries, including those of the brain^13^. Cerebral babesiosis, which occasionally develops in *B. bovis* infections, is manifested by hyperesthesia, nystagmus, circling, head pressing, aggression, convulsions, and paralysis; these signs may or may not accompany other signs of acute babesiosis^13–15^. The parasites in blood or tissue smear stained with Giemsa are frequently used to diagnose Babesia piroplasms. Thick films can assist in detecting small numbers of parasites, while thin films are ideal for species identification. However, fluorescent dyes such as acridine orange and immunostaining approaches have been described^14^.

Ticks are one of the most common ectoparasites in the world and are the major carriers of animal pathogens, especially in the tropical and subtropical regions of the world. As a parasite, ticks transmit tick-borne pathogens and cause anemia, irritation, skin abrasions, tick paralysis, udder and teat injury, and bite sores, which predispose to secondary bacterial infections^16,17^. Ticks and tick-borne pathogens in cattle have been studied in several regions of Ethiopia, and various species of ticks from the genera *Amblyomma, Rhipicephalus (Boophilus), Rhipicephalus, Hyalomma*, and *Hemaphysalis* have been identified^18–26^. Domestics and wild animals are infested by more than 60 tick species, 33 of which are considered major parasites of livestock^27^.

Despite their catastrophic effect on cattle and other livestock by diminishing the productive performance of the affected animals, livestock illness, particularly bovine babesiosis and the vector tick, has received insufficient attention in Ethiopia today^28^. Thus, the objective of this study was to determine the prevalence and associated risk factors for babesia infection and ixodid ticks of cattle in the Dasenech and Salamago District, southern Ethiopia.

## Methods

### Study sites

The study was conducted in the Salamago and Dasenech district of the South Omo Zone, the Southern Nation Nationalities and Peoples Regional State (SNNPRS) of Ethiopia, and it is located 110 km from Jinka, the city of the South Omo zone, and 597 km from Hawassa, the city of SNNPRS. The district covers approximately 451,120 km^2^. According to the Salamago Wasteal and Rural Development Office (2019/2020), the climatic conditions of the Wadal range from arid to subhumid. The average temperature and humidity of the study area are 32 °C and 977 mmHg, respectively. Grass, bushland, and some domestic trees, such as acacia, were found in study area^29^.

Dasenech district is located 206 km from zone capital Jinka, 642 km from the region capital Hawassa and 956 km from Addis Ababa and found near the Kenyan border. This area has latitudes and longitudes of 4° 45′-4° 99′ N and 35° ‘81’–36° 41′ E, respectively. Dasenech is found in the Omo Delta, which is an incredibly dry region with a temperature ranging up to 35 °C and cattle are central to the lives of the community. The areas have livestock populations of 640,500 cattle, 224,537 sheep, 282,427 goats, 22,400 donkeys, 350 camels, and 23,412 poultry. The woreda practice is predominantly pastoralism with little rainfed and irrigated agriculture^1^.

### Study animals

The study animals were indigenous cattle of both sexes, various age groups, body conditions, and various hair coat colors that were managed under an extensive management scheme with communal herding. The age of animals was grouped as young (1–2 years), adults (3–5 years), and old (> 5 years) according to the classification method used by Bitew et al.^30^, while body condition scores of animals were classified as emaciated (poor), moderate (medium), and good based on anatomical parts and the flesh and fat cover at different body parts^31^. Moreover, the study animals were categorized into five classes of white, red, black, and mixed coat colors to determine whether the coat color of the animal had any effect on the occurrence of the disease^32^.

### Study design

A cross-sectional study was conducted from September 2019 to August 2020 to determine the prevalence of Babesia infection and ixodid ticks of cattle and their associated risk factors in the Dasenech and Salamago Districts.

### Sampling method and sample size determination

The sites were selected based on the available cattle population and ease of transportation and environmental conditions, and selected sites (Peasant Association) were identified and selected based on convenience. The study cattle were selected by a simple random sampling method from each locality. The sample size of this parasitological survey was measured with a 95% confidence interval and 5% precision, and an estimated or expected prevalence was calculated using Thrusfield^33^.

$$ {\text{n}} = \frac{{{1}.{96}^{{2}} \;{\text{Pexp}}\;\left( {1 - {\text{Pexp}}} \right)}}{{{\text{d}}^{2} }} $$

where n = required sample size, Pexp = expected prevalence (50%), and d = desired absolute precision.

Accordingly, as per the predetermined parameters, the sample size computed was 384 cattle. To increase the precision of the study, a total of 470 cattle were sampled during the study. Moreover, the prevalence for each tick species was calculated as P = d/n × 100, where p = the prevalence, d = number of animals that tested positive for particular tick genera, and n—the total number of animals collected from animal species^34^.

### Sample collection and processing

All methods were performed in accordance with the best practice guidelines and regulations for veterinary care that were approved by the Wolaita Sodo University of Research Ethics and Review Committee. Moreover, the cattle owners were informed about the purpose of the study.

### Determination of parasitemia and packed cell volume (PCV)

The blood samples of 470 cattle were collected from the jugular vein and ear vein after disinfection with 70% alcohol for hematological analysis. Blood film examination was performed with Giemsa staining procedures, and microscopic examination of slides was conducted according to^35^. Blood samples were taken from the ear veins with piercing with a sterile lancet and then collected with heparinized capillary tubes after thin and thick blood smears were made and labeled. The slide was then air-dried and immediately fixed with absolute methyl alcohol for 3 min, and then, the smear was stained with 10% Giemsa stain for 45 min. Finally, a minimum of 50 fields of each stained blood smear was thoroughly examined under a compound microscope using oil immersion according to Foreyt^36^, to determine parasitemia and identify species of parasite based on a reference set by Soulsby^37^, and Moretti et al.^38^. Parasitemia was detected over Hemacolor^®^-stained thin blood smears by counting at least 1000 red blood cells and noting the number that was infested with Babesia.

Blood collected by jugular venipuncture in 10% EDTA-containing Vacutainer tubes was subjected to estimation of packed cell volume (PCV) using microhematocrit centrifugation. The microhematocrit capillary tubes (up to ¾) were filled with blood with a sealed outermost end. The blood specimens were centrifuged at 12,000 rpm for 5 min using a hematocrit centrifuge^35^. After centrifugation, the capillaries were put into the hematocrit reader for hematocrit (PCV) measurements, and then cattle were classified as anemic based on a modification of the classification criteria of Reyers et al. (1998) cited by Jacobson^14^ combined with that of Tvedten and Weiss^30^. Cattle with PCV of less than 24% were considered severely anemic, and those with PCV > 46% were considered nonanemic.

### Tick collection and identification

The entire body surface of the cattle was inspected for the presence of ticks. After fully restraining the animals, all visible adult tick species were removed by gloved hands and using forceps holding the base capitulum so as not to lose the mouthparts of the ticks. Ticks from each animal were collected and kept in separate prelabeled universal bottles containing 70% ethyl alcohol until identification. Ticks were counted and subsequently identified to the genus level by using a direct stereomicroscope using key morphological characteristics, i.e., size of mouthparts, the color of the body, leg color, presence or absence of the eye, the shape of scutum, body, coxae one, festoon, and ventral plates were considered as described by Walker et al.^39^ under a stereoscopic microscope.

### Data management and statistical analysis

Data collected during the study period were stored in Microsoft Excel spreadsheets and analyzed using statistical software called STATA version 13 for Windows (Stata Corp. College Station, USA). Descriptive statistics (frequencies and percentages) were used to determine the prevalence of babesia infection and tick infestation in cattle. The overall prevalence of babesia infection and ticks was determined by dividing the number of positive animals by the total sample size and was expressed as a percentage. The chi-square test and multivariate logistic analysis were used to examine the relationship between potential risk factors, including origin, age, sex, body condition, season, and the occurrence of both Babesia infection and tick infestation. Effects were reported as statistically significant in all cases if the value is less than 0.05 at a 95% confidence interval (CI).

### Ethics approval and consent to participate

Ethical approval for this research was obtained from the Wolaita Sodo University Research Ethics and Review Committee. Before collecting samples, informed consent was obtained from the cattle owners to take samples from their cattle and adopt strict hygienic measures.

## Results

### Frequency of babesiosis in cattle of the study area

**Figure 1:**
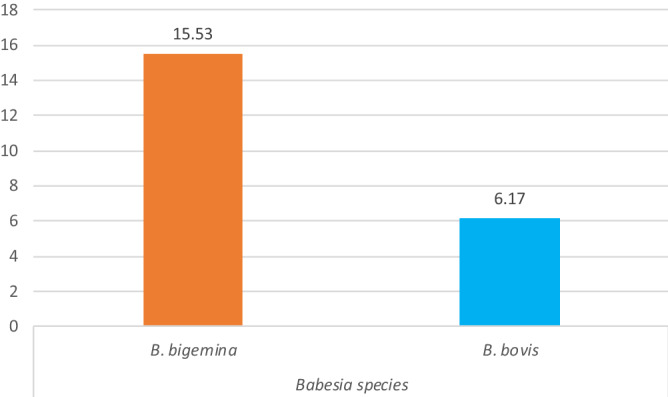
Proportion of babesia in the study districts.

Among 470 cattle studied for tick-transmitted hemoparasites, 102 (21.7%) were found to be infected with Babesia parasites. The *Babesia* species identified in the current study were *Babesia bigemina* and *B. bovis,* with an overall prevalence of 15.53% (73/470) and 6.17% (29/470), respectively (Fig. 1).

### Factors associated with the occurrence of babesia infection

In this study, a high prevalence of Babesia infection was noted in Salamago (50.9%) district, adult age (66.7%) group, male (54.9%) in sex, and poor body conditioned (68.6%) cattle compared with their respective groups. Moreover, no difference in the prevalence of babesia infection was observed in the wet (50%) and dry seasons (50%). Among putative risk factors, tick infestation and season of the year showed a statistically significant difference (*p* < 0.05) with the occurrence of babesia infection. On the other hand, there was no statistically significant variation (*p* < 0.05) between age, sex, and body condition score of cattle and the occurrence of babesia infection on both chi-square and multivariate logistic regression analysis (Table 1).

**Table 1:** Multivariate logistic regression of host factors and prevalence of bovine babesiosis.

Risk factor	Category	No. of Infected animals	Prevalence (%)	AOR	95% C. I. [lower–upper]	Chi-square (X^2^)	*p* value
Age	Young	19	18.6	Ref	Ref	2.017	0.37
	Adult	68	66.7	1.013	0.25–4.09		
	Old	15	14.7	1.63	0.28–9.46		
Sex	Male	56	54.9	Ref	Ref	0.117	0.73
	Female	46	45.1	1.56	0.52–4.68		
Body Condition Score	Good	22	21.6	1.30	0.25–6.76	3.69	0.16
	Moderate	10	19.8	1.50	0.37–6.12		
	Poor	70	68.6	Ref	Ref		
Season	Dry	51	50	Ref	Ref	4.96	0.026
	Wet	51	50	0.74	0.23–2.35		
Tick infestation	Present	97	95.1	2.22	–	8.71	0.003
	Absent	5	4.9	Ref	Ref		
Origin	Salamago	52	50.9	Ref	Ref	0.652	0.419
	Dasenech	50	49.1	0.50	0.17–1.51		

### Determination of the babesia infection status and mean PCV in study cattle

In the current study, the level of babesia infection status was determined by using thin and thick blood smears. Approximately 95% of cattle were parasitemic, and the remaining 5% were aparasitemic up on thin and thick blood smears, respectively. The mean PCV of anemic cattle due to babesia infection was 21.49% (CI = 21.09–21.89%). However, a statistically significant association (t = 29.67, *p* = 0.0001) was observed between the PCV of anemic and nonanemic cattle and the occurrence of babesia infection (Table 2).

**Table 2:** Mean PCV of infested and noninfested animals found in Dasenech and Salamago Districts.

PCV	No. examined	Mean PCV ± SD	t value	95% Conf. interval	*p* value
Anemic	102	21.49 ± 2.56	29.67	21.09–21.89	0.0001
Nonanemic	368	28.29 ± 2.23		28.04–28.54	
	470	26.01 ± 3.98		25.65–26.37	

### The overall prevalence of the ixodid tick

The overall prevalence of tick infestation in the current study was 53.8% (253/470). A high frequency of tick infestation was observed in Dasenech (54.9%) compared to Salamago (45.1%). The infestation of the ixodid tick during the wet season (50.6%) was found to be higher than that during the dry season (49.4%). Moreover, a statistically significant association (*p* < 0.05) was observed with the occurrence of ixodid ticks among address and season of the year (Table 4). However, no statistically significant variation (*p* > 0.05) was observed with the occurrence of the ixodid tick among other putative risk factors, such as age, sex, and body condition score, of the study cattle (Table 3).

**Table 3:** Prevalence of ixodid tick and its associated risk factors.

Risk factors	Tick infested animal	Prevalence (%)	Chi-square	*p* value
**Origin**				
Salamago	114	45.1	12.34	0.0001
Dasenech	139	54.9		
**Age**				
Young	45	17.8	0.275	0.872
Adult	179	70.7		
Old	29	11.5		
**Sex**				
Male	133	52.6	0.695	0.695
Female	120	47.4		
**Body condition score**				
Poor	180	71.2	1.15	0.564
Moderate	31	12.3		
Good	42	16.6		
**Season**				
Dry	125	49.4	18.35	0.0001
Wet	128	50.6		
Total	253	53.8		

### The prevalence of ixodid tick genera identified in the study district

The current study identified four different tick genera with a high prevalence, Amblyomma (37.5%), followed by Hyalomma (22.1%), Rhipicephalus (Boophilus) (21.3%), and Rhipicephalus (18.9%). Moreover, among putative risk factors, the origin of animals showed a statistically significant difference (*p* < 0.05) with the occurrence of *Amblyomma* spp. and *Hyalomma* spp. Although *Amblyomma* spp. and *Rhipicephalus* spp. showed a statistically significant difference (*p* < 0.05) with the season (Table 4).

**Table 4:** The ixodid tick genera identified in the study district.

Risk factors	Ixodid ticks species							
	*Amblyomma*		*Rhipicephalus (Boophilus)*		*Hyalomma*		*Rhipicephalus*	
	Freq (%)	X^2^ (*p* value)	Freq (%)	X^2^ (*p* value)	Freq (%)	X^2^ (*p* value)	Freq (%)	X^2^ (*p* value)
**Origin**								
Salamago	41 (43.1)	8.63 (0.003)	25 (46.2)	1.26 (0.26)	20 (35.9)	12.1 (0.0001)	23 (48.1)	0.74 (0.387)
Dasenech	54 (56.8)		29 (53.7)		36 (64.1)		25 (51.9)	
**Age**								
Young	17 (17.8)	0.65 (0.721)	12 (22.2)	2.85 (0.24)	9 (15.7)	0.33 (0.847)	5 (10.3)	3.16 (0.205)
Adult	68 (71.5)		34 (62.9)		41 (74.1)		39 (79.2)	
Old	10 (10.5)		8 (14.8)		6 (10.1)		4 (10.3)	
**Sex**								
Male	49 (51.5)	0.39 (0.530)	28 (51.8)	0.34 (0.55)	27 (48.3)	1.14 (0.285)	28 (58.4)	0.94 (0.333)
Female	46 (48.4)		26 (48.1)		29 (51.6)		20 (41.5)	
**Body condition score**								
Good	16 (16.8)	0.91 (0.633)	10 (18.5)	0.38 (0.82)	8 (14.6)	2.76 (0.251)	11 (23.3)	3.70 (0.157)
Moderate	12 (12.6)		7 (12.9)		6 (8.99)		6 (10.3)	
Poor	67 (70.5)		37 (68.5)		42 (76.4)		31 (66.2)	
**Season**								
Wet	60 (63.1)	48.2 (0.0001)	23 (42.5)	0.04 (0.84)	27 (48.3)	2.83 (0.092)	30 (62.4)	18.4 (0.0001)
Dry	35 (36.8)		31 (57.4)		29 (51.6)		18 (37.6)	
Over all prevalence	95 (37.5)		54 (21.3)		56 (22.1)		48 (18.9)	

The odds of cattle infested by a tick in the Dasenech area were 2.38 times higher (CI, 1.60–3.55) when those cattle infested by a tick from the Salamago area were kept constant. When dry seasons were held constant, the odds of cattle being affected by ixodid ticks increased by 0.37 times (CI, 0.248–0.561). However, a statistically significant association (*p* < 0.05) was observed between tick infestation and season and origin. However, there was no statistically significant association (*p* > 0.05) between sex, age, and body condition score of the animal and the occurrence of ixodid tick (Table 5).

**Table 5:** Multivariate logistic regression of host factors and prevalence of ixodid tick.

Risk factors	OR	St. Err.	95% Conf. interval	*p* value
**Origin**				
Dasenech	2.38	0.484	1.60–3.55	0.0001
Salamago	Ref	Ref	Ref	Ref
**Age**				
Adult	0.90	0.238	0.536–1.511	0.691
Old	1.03	0.383	0.497–2.135	0.936
Young	Ref	Ref	Ref	Ref
**Sex**				
Female	1.20	0.234	0.817–1.761	0.351
Male	Ref	Ref	Ref	Ref
**Body condition score**				
Poor	0.91	0.244	0.536–1.538	0.720
Moderate	0.66	0.192	0.378–1.172	0.159
Good	Ref	Ref	Ref	Ref
**Season**				
Wet	0.37	0.077	0.248- 0.561	0.0001
Dry	Ref	Ref	Ref	Ref

### Tick distribution across the animal’s body

A total of 8040 ticks were collected from various body parts of cattle in the current investigation from a total of 253 tick-infested cattle out of 470 investigated animals. Accordingly, the highest proportions of ticks were collected from the scrotum/udder (32.10%), followed by the dewlap and neck (22.06%), anus, and vulva (20.14%), and head and ear (13.84%). Additionally, the majority of *Rhipicephalus* species were collected from scrotum/udder (32.91%) and dewlap and neck (27.09%), although a high number of *Amblyomma* species were collected from scrotum/udder (43.9%) and brisket (29.03%). On the other hand, *Rhipicephalus (Boophilus)* species were found around the anus and vulva (35.01%) as well as the head and ear (22.75%), whereas *Hyalomma* species were the least collected type of tick genera during the current study and were mostly found around the anus and vulva region (29.50%) and brisket (23.40%) (Table 6).

**Table 6:** Proportion and tick distribution in various body parts within the study districts.

Body part	*Rhipicephalus*		*Amblyomma*		*Rhipicephalus (Boophilus)*		*Hyalomma*		Total	Proportion (%)
	No.	%	No.	%	No.	%	No.	%		
Head and ear	806	14.39	37	3.62	245	22.75	25	7.59	1113	13.84
Dewlap and neck	1520	27.09	79	7.72	126	11.69	49	14.89	1774	22.06
Brisket	248	4.41	297	29.03	46	4.27	77	23.40	668	8.32
Belly and back	141	2.51	65	6.35	67	6.22	12	3.65	285	3.54
Scrotum/udder	1847	32.91	449	43.90	216	20.06	69	20.97	2581	32.10
Anus and vulva	1049	18.96	96	9.38	377	35.01	97	29.50	1619	20.14
Total	**5611**	**100.00**	**1023**	**100.00**	**1077**	**100.00**	**329**	**100.00**	**8040**	**100.00**

Significant values are in [bold].

## Discussion

*Babesia* spp. and ticks are major constraints of livestock production that have a worldwide distribution with greater economic importance through direct and indirect effects on their hosts in tropical and subtropical regions by Criado-Fornelio et al.^40^, Rahman et al.^41^; Filia et al.^42^, and have been reported in Ethiopia by Sitotaw et al.^43^; Solomon and Tanga^36^. In this study, the overall prevalence of Babesia infection was 21.7%, which was compatible with the reports of Lemma et al.^44^ and Simking et al.^37^, who reported a prevalence of 23% in Jimma town and 26.6% in Salakpra Wildlife Sanctuary, Kanchanaburi Province, respectively. However, this finding was higher than the reports of Hamsho et al.^4^; Alemayehu^38^; Worku^45^; Solomon and Tanga^46^; Ayaz et al.^47^; Ahmad and Hashmi^48^; Shane et al.^49^; Waktole et al.^50^; Wodajnew et al.^51^; Ola-Fadunsin et al.^52^ and Sitotaw et al.^43^, who reported a prevalence of 16.9% in Teltele district, 12% in Bariso, 11.4% in Arsi, 6.51% in Alle district, 9.9% in Kohat and Karak District, 6.6% in Lahore, 6% in Tiyo District, 3.64% in Meki and Batu Towns, 1.5% in Assosa district, 1.2% in Nigeria and 0.3% in Debre-Zeit, central Ethiopia. In contrast, the current study's findings were lower than those reported by Rahman et al.^53^ and Mohammed and Ebied^54^, who found prevalence rates of 42% in Malaysia and 40% in Benha, Qalubia Governorate, northeastern Egypt, respectively. This variation in babesiosis prevalence in cattle could be attributed to differences in the study area, the use of acaricides during tick infestation, the sensitivity of diagnostic tests used, proper use of antiparasitic drugs, cattle management systems in the focus area, and sampling seasons of the year, as well as the land use of the area, the distribution of infected vectors, and the accessibility of animals to wildlife reserves and parks and forest areas containing tick vectors, which vary among the study areas^55,56^.

The overall prevalence of *Babesia species* was 15.53% (*B. bigemina*) *and* 6.17% (*B. bovis*)*.* This result corresponded with the former description of Solomon and Tanga^46^, who reported a prevalence of 6.51% for *Babesia bovis* from Alle District, Southwestern Ethiopia. Contrary to this finding, a lower prevalence was reported by Waktole et al.^50^; Wodajnew et al.^51^, and Abdela et al.^57^, who reported a prevalence of 3.38% (*B. bigemina*) *and* 0.26% (*B. bovis*) from Meki and Batu towns*,* 0.248% (*B. bigemina*) and 1.24% (*B. bovis*) from Assosa Ethiopia, and 9.8% (*B. bigemina*) and 2.2% (*B. bovis*) from Jimma town, respectively. However, a higher prevalence was reported by Tembue et al.^58^; Lemma et al.^44^; Rahman et al.^41^; Hamsho et al.^4^, who reported a prevalence of 78.8% (*B. bovis*) and 76.0% (*B. bigemina*) from southern Mozambique*,* 60.8% (*B. bovis*) and 39% (*B. bigemina*) from Jimma town in southwestern Ethiopia, 17% (*B. bovis*) and 16% (*B. bigemina*) from Malaysia, and 9.9% (*B. bovis*) and 7.03% (*B. bigemina*) from Borana, respectively. Different investigations have also revealed that cattle infected with *B. bovis* remain carriers for long periods, while those infected with *B. bigemina* remain carriers for only a few months^13,56^. According to Mekonnen^59^, the widespread nature of *B. bigemina* in Ethiopia can be associated with its vector *R.(B). decoloratus,* which is the most widespread one-host cattle tick in Ethiopia^59,60^. Moreover, the probability of mechanical transmission is low with *B. bovis* and high with *B. bigemina*^61^. Thus, the predominance of *B. bigemina* infection detected in this study is not surprising since the distribution of both *B. bovis* and *B. bigemina* is determined by the distribution of their tick vectors.

In the current study, a higher prevalence of bovine babesiosis was noted in adult age (66.7%), followed by young (18.6%) and old age cattle (14.7%). In contrast, Lemma et al. (2015) from Jimma town in southwestern Ethiopia, Waktole et al.^50^ from the Maki and Batu areas, and Ayaz et al.^47^ from Pakistan consistently reported a high prevalence of Babesia infection in old age cattle, with prevalences of 27%, 7.5%, and 13.4%, respectively. However, Amorim et al.^62^ identified that calves were more susceptible to Babesia species than adult cows, which was also opposed to the present finding. Young animals, particularly calves under the age of six months, are more likely to be resistant to minor infestations than older animals because they develop acquired immunity by feeding Colostrum of the dam^63^. On the other hand, the low frequency in young animals is due to limited grazing by young animals, which may reduce the likelihood of exposure to this vector^64^.

The prevalence of babesia infection in males (54.9%) was slightly higher than that in female (45.1%) cattle. This finding was in agreement with the report of Solomon and Tanga^46^ and Waktole et al.^50^, who found a high prevalence of babesiosis in male cattle (7.09%) and 4.4% compared to females*.* Nevertheless, this result disagrees with the report of Kocan et al.^65^, who found a higher prevalence of babesiosis in females (11.2%) than in male cattle (6.96%). The higher prevalence of babesia infection in male animals could be linked to their being employed for farming purposes (plowing), which could cause stress and make them immunocompromised, as well as being kept out of the door, where they could be infected by a tick vector.

The prevalence of the disease based on the body condition of the animals was 21.6%, 19.8%, and 68.6% for good, medium, and poor body condition scoring, respectively, which was comparable with reports described by Hamsho et al.^4^; Wodajnew et al., 2015 and Sitotaw et al., 2014. This could be because animals with poor body conditions have lower immunity that encourages infection of the animal by different organisms, such as Babesia.

The odds of babesia infection in the wet season was 0.74 times when the dry season was kept constant and showed a statistically significant difference (*p* < 0.05) with the occurrence of babesia infection. Similarly, Kamani et al.^64^; Solomon and Tanga^46^, and Waktole et al.^50^ reported a high prevalence of Babesia infection in the wet season compared to the dry season. In the seasonal prevalence of the disease, the disease was more prevalent in wet conditions, probably the reason behind this trend may be correlated to the seasonal activities of the hard ticks, which are more abundant on the days after the rain peaks, thus resulting in a higher incidence of babesia infection, as reported by Soulsby^66^.

The availability of vectors is a potential risk factor for Babesia infections^67^. The multivariate logistic regression analysis showed that the risk of babesiosis was significantly (*p* < 0.005) higher in tick-infested (95.1%) cattle than in nontick-infested (4.9%) cattle. A similar observation was reported by Costa et al.^68^. The presence of Babesia infection in cattle in the study area is broadly related to the presence of suitable vectors (ticks).

In the current study, there was a statistically significant (*p* = 0.0001) variation in the mean PCV value between infected cattle (21.49%) and noninfected cattle (28.29%), which was in line with the previous report by Waktole et al.^50^ and Sitotaw et al.^43^. This suggests that anemia is caused by the blood suck of ticks or babesiosis since the parasite invades red blood cells to cause hemolysis. Moreover, anemia develops as a result of blood hemolysis, and hemolysis occurs due to mechanical damage by trophozoites to RBCs when multiplied by binary fission, phagocytosis of infected RBCs by the host immune system, and toxic substances secreted by the parasites^69^.

The overall prevalence of tick infestation in the current study was 53.8% (253/470). This was lower than the previous findings of Ayana et al.^70^; Mesfin et al.^71^; Kumisa et al.^72^; Abera et al.^5^; Meaza et al.^73^; Shichibi et al.^74^; Kemal et al.^22^; Teshome et al.^24^; Wogayehu et al.^75^; Tamerat et al.^76^; Alemu et al.^77^; Wolde and Mohamed^78^; Meseret et al.^79^; Wasihun and Doda^80^ and de Castro^81^, who reported prevalences of 89.89%, 89.1%, 68.8%, 97.8%, 91.7%, (88.54%, 91.50%, 78.84%), 75.7%, 70.31%, 68.12%, 82%, 81.25%, 65.5%, 59.6%, 61% and > 80% from different parts of the country, respectively. On the other hand, the overall prevalence of tick infestation in the current study was higher than the previous reports described by Solomon and Tanga^46^; Fesseha and Mathewos^21^; Tiki and Addis^82^; Waktole et al.^50^ and Zelalem et al.^26^, who reported a prevalence of 36.19% in Alle district, 42.2% in Hosana district, 25.64% in Holeta district, 29.4% in Maki and Batu area, and 38% in the Chiro district, respectively. This may be due to differences in study area coverage and environment. This can also be due to factors such as animal health practices, temperature, and humidity that promote the survival and growth of ticks during their development, acaricide application as a control method, a low level of awareness of farmers, and the proportion of ticks^83,84^.

In this study, a high frequency of tick infestation was observed in Dasenech (54.9%) compared to Salamago (45.1%), with a statistically significant association (*p* < 0.05) with the occurrence of Ixodidae ticks. This could be due to the differences in the agroclimatic conditions of the study areas and the season of sample collection. It was reported that tick activity can be influenced by rainfall, altitude, season, and atmospheric relative humidity^19^. In addition, a large livestock population and herd size contribute to tick infestation, as ticks can easily obtain access to hosts and complete their life cycle to continue rapidly, and poor veterinary service and less attention are given to cattle management practices employed by herders, which might also pave the way for tick infestation.

In terms of the sex of the host, the prevalence of ixodid ticks by sex revealed that males (52.6%) were more infected than female (47.4%) cattle, with a statistically insignificant difference (*p* > 0.05). This finding agrees with the finding of Wasihun and Doda^28^, who reported higher infestation in male animals than in females. However, contrary to our findings, Abdeta et al.^18^ and Kassa and Yalew^22^ reported a higher prevalence in female animals (68% and 18.8%) compared to males (82.06% and 14.23%), respectively. This could be attributed to female cattle being kept in the house with proper management for dairy purposes, while male cattle grazed on a field all day can be exposed to tick infestation^85^.

Cattle with poor (71.2%) and good (16.6%) body condition scores showed a high prevalence of tick infestation compared to moderate body-conditioned cattle (12.3%), which did not show a statistically significant (*p* > 0.05) difference with the occurrence of tick infestation. Similarly, Kassa and Yalew^86^ and Shiferaw and Onu^34^ reported a statistically insignificant correlation (*p* > 0.05) between the existence of Ixodidae ticks and the animal’s body condition score. On the other hand, Tiki and Addis^82^ reported a statistically significant difference (*p* < 0.05) between body condition scores and the existence of ticks. The high infestation rate of hard ticks in poor body condition scores compared to moderately conditioned animals could be due to the less resistant behavior of weak animals to tick infestation and lack of enough body energy to build resistance^36,87^.

The proportion of tick infestation was higher in the adult (70.7%) than in the old (11.5%) and young (17.8%) age groups, with a statistically insignificant (*p* > 0.05) difference in the occurrence of tick infestation. This finding was strengthened by the findings of Desalegn et al.^88^, who reported higher infestation in adult cattle, and agrees with the findings of different authors Kemal et al.^22^, Yalew et al.^89^, Ayana et al.^70^, Fesseha and Mathewos^21^, Shichibi et al.^74^, Meaza et al.^73^, Ayana et al.^90^, and Okello-Onen et al.^91^, who reported a higher proportion of adult cattle from different parts of the country. A higher proportion may be due to the long-distance movement of adult cattle to search for food, which increases the chance of contact with ticks and low immunity in older animals.

In the current study, *Amblyomma* was found to be the most abundant tick genus, followed by *Hyalomma*, *Rhipicephalu (Boophilus)*, and *Rhipicephalus*. This finding was strengthened by Kassa and Yalew^86^, Kemal et al.^22^, Ayalew et al.^19^, Wasihun and Doda^80^ and Yehualashet et al.^92^, who reported that *Amblyomma* was the most common and widely distributed cattle tick in Ethiopia and African countries. In contrast to this result, Ayana et al.^70^ and Waktole et al.^50^ reported *Rhipicephalus* as the dominant tick genus. In addition, according to Shane et al.^49^, *Boophilus* was identified as the main tick species in Tiyo District. This may be due to the different seasons in which the survey was conducted^5,81^.

In the current study, a total of 8040 ticks were collected from various parts of the animal body. The highest tick frequency was gathered from the scrotum/udder (32.10%), followed by dewlap and neck (22.06%), anus and vulva (20.14%), and head and ear (13.84%). Additionally, the majority of *Rhipicephalus* species were collected from scrotum/udder (32.91%) and dewlap and neck (27.09%). However, Ayana et al.^70^ from the Yabello district reported a large number of ticks from the head and ear (34.57%) region. Furthermore, *Amblyomma* species were collected in a high percentage from scrotum/udder and brisket, whereas *Rhipicephalus* species were collected from head and ear and anus and vulva, *Hyalomma* species were collected from scrotum/udder, anus and vulva and dewlap and neck, and *Boophilus* species were collected from anus and vulva part of the body.

## Conclusion

The present study revealed a high prevalence of Babesia infection and ixodid ticks in cattle in the Dasenech and Salamago Districts. *Babesia bigemina and Babesia bovis* are the two most important Babesia species of cattle that were identified in the study area. A statistically significant difference (*p* < 0.05) was observed between season and tick infestation with the occurrence of Babesia infection. In this study, Ambylomma was found to be the most predominant tick genus, followed by Hyalomma, Rhipicephalus (Boophilus), and Rhipicephalus. Thus, seroprevalence and molecular studies to identify the predominant Babesia species and for the detection of Babesia in tick hemolymph for the identification of tick genera responsible for the occurrence of Babesia infection are crucial. Additionally, tailoring suitable and coordinated tick management methods using chemotherapy as well as strategic treatment to overt clinical cases of bovine babesiosis is critical.

## Data Availability

The datasets used and analyzed during the current study are available from the corresponding author on request.

## Abbreviations

AOR: Adjusted odds ratio
CSA: Central Statistical Authority
EDTA: Ethylene Diminetetra acetic acid
OIE: Office of des International Epizootics
PCV: Packed cell volume
RBC: Red blood cell
RPM: Revolution per minute
